# ENHANCE: a global collaborative research programme investigating the role of homeopathy in antimicrobial stewardship

**DOI:** 10.3389/frabi.2026.1892945

**Published:** 2026-07-30

**Authors:** Esther van der Werf

**Affiliations:** 1 Homeopathy Research Institute, London, United Kingdom; 2 Centre for Academic Primary Care, Bristol Medical School, University of Bristol, Bristol, United Kingdom

**Keywords:** antimicrobial resistance, antimicrobial stewardship, ENHANCE, homeopathy, one health, primary care, TCIM

## Abstract

Antimicrobial resistance (AMR) is a leading global health threat. Primary care accounts for approximately 80% of human antibiotic use, making novel non-antibiotic strategies for common self-limiting infections an urgent need. Traditional, Complementary and Integrative Medicine (TCIM), including homeopathy, is widely used worldwide; the existing literature on homeopathy suggests a directional signal of potential reductions in antibiotic use in both human and veterinary settings, though the evidence base remains heterogeneous and methodologically limited. The ENHANCE (rEsearch programme oN Homeopathy and Antimicrobial resistaNCE) international collaborative Programme was established in 2023 to systematically evaluate homeopathy’s role in antimicrobial stewardship (AMS) through five integrated workstreams: 1) population studies, 2) AMR literature monitoring, 3) Condition-specific systematic reviews, 4) Core outcome set development, and 5) Primary evidence generation. Completed outputs include systematic reviews for otitis media (2024) and tonsillitis (2026), the first internationally agreed Core Outcome Set for acute otitis media (COS-AOM, 2025), and a nationally representative UK survey showing that homeopathic product use occurs in about 5% of the population (an estimated 2.7 million people), with 4% (around 2.2 million people) consulting homeopathic practitioners (2026). Ongoing work includes systematic reviews and core outcome sets for tonsillitis and sinusitis, and the design of pragmatic primary care trials and real-world evidence studies aligned with WHO- and One Health AMR and AMS strategies. ENHANCE provides the methodologically rigorous, internationally coordinated infrastructure needed to determine whether homeopathy can meaningfully contribute to antibiotic-sparing stewardship.

## Introduction

Antimicrobial resistance (AMR) represents one of the most urgent threats to global health, with an estimated 4.95 million deaths associated with bacterial AMR in 2019 (Antimicrobial Resistance Collaborators, 2022). The WHO Global Action Plan on AMR identifies optimising antimicrobial use as a strategic priority, particularly in primary care settings where approximately 80% of human antimicrobial use occurs (World Health Organization, 2015). In the UK, the 5-year AMR Strategy (2019-2024) and its successor 20-year Vision emphasise reducing inappropriate antibiotic prescribing while maintaining effective infection management (UK Department of Health and Social Care, 2019; UK Department of Health and Social Care, 2024).

Common primary care infections, particularly respiratory tract infections, acute otitis media (AOM), and uncomplicated urinary tract infections, account for a major share of antibiotic prescribing despite evidence that many cases resolve spontaneously (Pouwels et al., 2018). For example, 78% of AOM cases in children resolve without antibiotics, yet it remains a leading indication for paediatric antibiotic prescriptions globally (Venekamp et al., 2023). This disconnect between clinical necessity and prescribing practice creates an opportunity for stewardship interventions, including the investigation of non-antibiotic therapeutic approaches.

Traditional, Complementary and Integrative Medicine (TCIM) represents a broad category of healthcare practices, with homeopathy among the most widely used in Europe. Worldwide, over 200 million people use homeopathy on a regular basis (Prasad, 2007), and 29% of the EU population use homeopathic medicines in their day-to-day healthcare (Commission of the European Communities, 1997). Recent UK data reveal that 4.9% of the population used homeopathic products and 4.1% consulted a homeopath over a 12-month period (van der Werf et al., 2026).

While the exact mechanism of action for homeopathic medicines is not yet fully understood, increasing evidence from physicochemical and biological studies supports their plausibility. A scoping review found 216 publications proposing different mechanisms, encompassing frameworks like complex systems theory, nanostructure models, and water organization hypotheses (Dombrowsky et al., 2025). Biological research further backs this, with a systematic review showing that 77% of foundational experiments demonstrated reproducible effects of homeopathic preparations in cell, animal, and plant models (Witt et al., 2007). Recent studies in neuronal cell lines (Lejri et al., 2025) and agrohomeopathy models (Ucker et al., 2022; Faedo et al., 2024) also confirm these findings. Notably, these *in vitro* and non-human studies exclude placebo effects as confounders, offering important context for interpreting clinical results.

## Homeopathy as a non-antibiotic strategy: evidence from human and veterinary medicine

The clinical evidence base for homeopathy is substantial, comprising 708 controlled clinical studies (Gaertner et al., 2023). A systematic review of meta-analyses capturing data from 182 randomised clinical trials found that five out of six meta-analyses demonstrated a statistically significant effect of homeopathy beyond placebo (Hamre et al., 2023). The most rigorous meta-analysis of individualised homeopathy (n=22 trials) reported that it is 1.5–2.0 times more likely to be beneficial than placebo, with stronger effects in higher-quality trials (Mathie et al., 2014), while a complementary meta-analysis indicated moderate positive effects for non-individualised homeopathy (Mathie et al., 2017). Homeopathy also has a favourable safety profile, with meta-analyses reporting adverse event rates comparable to placebo and serious adverse event rates significantly lower than comparable conventional treatments (Stub et al., 2016; Stub et al., 2022).

Homeopathy has been investigated as a means of reducing antibiotic prescribing across a range of human and veterinary infectious conditions. In acute otitis media, non-randomised controlled trials (nRCT) and pragmatic randomised controlled trials (RCT) have reported substantially lower antibiotic use in homeopathically treated children alongside comparable or faster symptom resolution (Friese et al., 1997; Wustrow and Otovowen Study, G., 2004; Taylor and Jacobs, 2014; Perry et al., 2024; Varanasi et al., 2025). In recurrent tonsillitis, an international pragmatic RCT found that adjunctive homeopathy reduced acute antibiotic use from 58.2% to 37.0% (p=0.0008) (Palm et al., 2017), and in recurrent urinary tract infections in spinal cord injury patients, classical homeopathy significantly reduced infection frequency (p<0.0001) with secondary reductions in antibiotic use (Pannek et al., 2019). At population level, the French EPI3 cohort study found 57% lower antibiotic consumption among patients of homeopathic general practitioners compared with conventional practitioners (OR = 0.43; p<0.001), with comparable clinical outcomes (Grimaldi-Bensouda et al., 2014). Registry-based data from Germany, a retrospective NICU analysis, and an observational study of UK NHS GP surgeries have similarly identified lower antibiotic prescribing in integrative medicine settings.

Evidence in veterinary medicine is more heterogeneous. In dairy cattle, four RCTs in clinical mastitis have produced mixed results, with antibiotics superior to homeopathy in pathogen-positive cases but comparable bacteriological cure in culture-negative cases (Hektoen et al., 2004; Werner et al., 2010; Ebert et al., 2017; Keller and Sundrum, 2018), and a non-inferiority trial of homeopathy versus antibiotic teat sealant for dry-cow mastitis prevention has also been reported (Klocke et al., 2010). Herd-level work in organic dairy systems has shown substantial antibiotic-treatment reductions associated with multicomponent programmes in which homeopathy was the principal modality, though these reductions cannot be attributed to homeopathy alone (Ivemeyer et al., 2008). In swine, homeopathy has been shown superior to low-dose antibiotic metaphylaxis for respiratory disease prevention (Albrecht and Schutte, 1999), and the strongest positive veterinary trial found homeopathic treatment via sows significantly reduced neonatal *E. coli* diarrhoea (p<0.0001) (Camerlink et al., 2010). A retrospective cohort study of turkey farms also reported markedly lower antibiotic-treatment days in homeopathy-oriented farms (Sonnenschein-Swanson et al., 2026).

The existing evidence does not yet meet the standard required for inclusion in antimicrobial stewardship guidelines, but the consistent directional signal across diverse settings justifies adequately powered, pre-registered, double-blind trials with antibiotic use as a primary outcome, complemented by real-world evidence (RWE) studies. Beyond AMR itself, the wider effects of antibiotics on the gut microbiome, as a sharp decline in bacterial diversity with recovery typically within weeks but persistent effects in some studies for 2–6 months (Elvers et al., 2020) strengthen the case for both antibiotic-sparing treatment strategies and microbiome-conserving prevention strategies.

Against this background, the ENHANCE (rEsearch programme oN Homeopathy and Antimicrobial resistaNCE) collaborative Programme was established in 2023 to systematically investigate homeopathy’s potential role in antimicrobial stewardship. Rather than advocating for or against homeopathy adoption, ENHANCE aims to generate high-quality evidence addressing specific research questions through the synthesis of existing data, developing new methodological tools for high-quality TCIM research, and conducting new primary research. The current paper describes ENHANCE’s research aims, structure and governance, methodological approach, completed studies, ongoing projects and future research directions.

## ENHANCE programme aims

The ENHANCE Programme has five overarching aims:

1. Synthesise existing evidence on the effectiveness of homeopathy in reducing antibiotic use across common primary care infections through condition-specific systematic reviews and meta-analyses
2. Standardise research methodology by developing internationally agreed Core Outcome Sets (COS), enabling cross-study comparisons and evidence synthesis
3. Characterise real-world TCIM use and its relationship to antibiotic prescribing behaviour at population level through national and international surveys
4. Generate primary evidence through adequately powered, pre-registered trials and real-world evidence studies with antibiotic use as a primary outcome
5. Generate research impact by translating findings into policy-relevant outputs that can inform antimicrobial stewardship guidelines within national and international One Health frameworks.

## Programme structure and governance

ENHANCE operates as a collaborative research programme that brings together investigators from UK universities and research institutes, international research networks, and international academic partners. The Programme is led and coordinated by the Homeopathy Research Institute (HRI), with most projects to date supported by researchers from the Bristol Medical School, University of Bristol, UK. International collaboration extends to European, Asian, and Australian Universities. **Appendix 1** lists the current ENHANCE collaborative researchers and their affiliations.

## The research is organised into five interconnected work streams

1. Population studies (survey of TCIM use patterns and attitudes)
2. Scientific monitoring of new publications on homeopathy and AMR/AMR stewardship
3. Systematic evidence synthesis (condition-specific systematic reviews and meta-analyses)
4. Research methodology development (core outcome sets)
5. Evidence generation (Randomised controlled trials and Real-World Evidence (RWE) studies in primary and community care).

## ENHANCE: completed work (published)

### Systematic review for acute otitis media

The Programme’s first major output was a comprehensive systematic review (PROSPERO CRD42022367188) examining homeopathy’s effectiveness in relieving symptoms and reducing antibiotic use in patients with otitis media, published in Heliyon in October 2024 (Perry et al., 2024).

This systematic review evaluated whether homeopathy can effectively relieve symptoms and reduce antibiotic use in patients with otitis media. Seven databases and four trial registries were searched for randomised and non-randomised controlled trials testing individualised or non-individualised homeopathy against placebo or standard care. Nine studies (7 RCTs, 2 non-RCTs) were identified comparing homeopathy with placebo (n=2) or standard care (n=7). 5 out of 9 studies reported on antibiotic consumption, 4 RCTs reported statistically significant outcomes favouring homeopathy for symptom scores, middle ear effusion, and antibiotic use. Meta-analysis revealed that add-on non-individualised homeopathy reduced filled antibiotic prescriptions by 46% (RR = 0.54, P = 0.07), though this was not statistically significant. The authors concluded that while individual RCTs show positive effects on symptom relief and antibiotic use reduction for patients with OM, heterogeneity across studies limits firm overall conclusions. Outcome heterogeneity was identified as the critical barrier to evidence synthesis, which directly informed the Programme’s next major initiative.

All systematic reviews followed the Cochrane Handbook for Systematic Reviews of Interventions (Higgins et al., 2023) with PRISMA 2020 reporting (Page et al., 2021) and were prospectively registered on PROSPERO.

### Systematic review for tonsillitis

The Programme’s next systematic review examined homeopathy’s effectiveness for patients with tonsillitis, published in the European Journal of Integrative Medicine in April 2026 (Perry et al., 2026).

This systematic review evaluated whether homeopathy could improve symptoms, reduce recurrence, and lower antibiotic use across acute, recurrent, and chronic tonsillitis presentations. Nine databases and four trial registries were searched for randomised and non-randomised controlled trials testing individualised (IH) or non-individualised homeopathy (non-IH) against placebo or active controls in patients of all ages. Five RCTs (seven publications) were identified, comparing homeopathy with placebo (n=4) or standard care (n=1); one used IH, one used both IH and non-IH, and three used non-IH. Two trials examined acute tonsillitis, two recurrent tonsillitis, and one chronic tonsillitis, with sample sizes ranging from 30 to 256. Individual RCTs showed that in acute tonsillitis, non-IH achieved short-term symptom improvement (reduced pain, throat inflammation, and tonsil size over six days); in recurrent tonsillitis, both IH and non-IH were associated with reduced infection recurrence, lower antibiotic consumption, fewer URTIs, and improved quality of life, with the strongest evidence for the standardised non-IH complex *SilAtro-5-90* (RR = 0.53 for recurrence, p<0.0001; RR = 0.64 for antibiotic use, p=0.001); in chronic tonsillitis, IH showed delayed but consistent improvements in symptoms, recurrence, healthcare utilisation, and quality of life by 2–3 months, with early outcomes favouring placebo. A cost-effectiveness analysis of *SilAtro-5-90* revealed an ICER of €156.64 per episode of acute tonsillitis prevented, with the intervention being cost-saving for patients with more than 3.33 previous episodes (Ostermann et al., 2021). No serious adverse events were reported. Meta-analysis was not possible due to substantial heterogeneity across populations, interventions, and outcomes, and the certainty of evidence (GRADE) was low for all outcomes.

While individual trials suggest that both IH and non-IH may provide symptom relief, reduce recurrence, lower antibiotic use, and enhance quality of life in tonsillitis, with no safety concerns, once again, methodological limitations and heterogeneity prevent definitive conclusions. Therefore, a Core Outcome Set for tonsillitis to support future evidence synthesis and clinical decision-making has been added to workstream 4 of ENHANCE.

### Core outcome set AOM

In response to the Otitis Media systematic review’s identification of outcome heterogeneity as a barrier to evidence synthesis, ENHANCE investigators developed the first internationally agreed Core Outcome Set for Acute Otitis Media (COS-AOM) for primary and community care studies. This work, published in BMC Primary Care in April 2025 (van der Werf et al., 2025), was guided by the COMET Initiative guidelines and employed a rigorous four-phase consensus methodology (Williamson et al., 2017). Phase 1 identified 51 candidate outcomes from three systematic reviews, which were condensed to 20 overarching outcomes. Phase 2 involved eight parents in a Public Involvement meeting to discuss the importance and relevance of these outcomes. Phase 3 gathered clinical perspectives through a ranking task completed by 28 healthcare professionals (11 GPs, 11 Traditional Complementary and Integrative Medicine professionals, and 6 pharmacists), achieving moderate agreement across all three groups. Phase 4 involved an International Steering Committee that finalised the Core Outcome Set through consensus discussions.

The COS-AOM comprises 10 outcomes in total: 8 acute outcomes (symptom severity, pain severity, pain duration, symptom duration, fever >38 °C, pain medication use, antibiotic use, and adverse events/serious complications) and 2 mid-to-long-term outcomes (recurrence/number of recurrences in 12 months, and presence of middle ear effusion/glue ear). All outcomes achieved 100% agreement from the steering committee. The COS-AOM provides standardised outcome measures enabling future meta-analyses and cross-study comparisons, directly addressing the barrier identified in the systematic review.

### National survey of TCIM use in the UK

A nationally representative survey examining patterns of TCIM use in the UK population was published in BMJ Open in January 2026 (van der Werf et al., 2026). This cross-sectional online survey investigated the prevalence and characteristics of TCIM, including homeopathy, use among UK adults. Data were collected from 1,559 participants (aged 18+ years) across England, Wales, Scotland, and Northern Ireland between May and October 2024, via a 94-item instrument, using purposive convenience sampling to achieve national proportional representation by national gender, age, and region. The survey covered demographics, health status, homeopathy product and practitioner use, patient-centred care experiences (Patient-Centred Care Scale), and disclosure of complementary medicine use to conventional practitioners (Complementary Medicine Disclosure Index).

The study found that 65.9% of UK adults used TCIM over a 12-month period, with 19.1% consulting a TCIM practitioner and 63.3% using TCIM products or practices. Of 1,559 eligible participants, 119 (7.6%) reported any homeopathy use in the past 12 months; 4.9% (an estimated 2.7 million people) used homeopathic products and 4.1% (around 2.2 million people) consulted a homeopath. Homeopathy ranked 4th in terms of both practitioner consultation and medicinal product usage amongst TCIM modalities in the UK.

Participants who consulted with a homeopath (n=64; 4.1%) also commonly visited a pharmacist (85.9%) or general practitioner (82.8%). These findings suggest that homeopathy mainly serves as a complementary therapy rather than an alternative one, with most users continuing to engage with conventional healthcare providers. Among those using both conventional and homeopathic products, concurrent use was generally infrequent.

Regarding antibiotic use, the study found that people were equally likely to use antibiotics regardless of whether they used TCIM, and there were no significant differences in their beliefs about whether antibiotics can treat colds or whether antibiotic use can cause side effects. However, people who use TCIM are statistically significantly less likely to believe that antibiotics can kill viruses. They are, however, more likely to believe that unnecessary antibiotic use does not affect antibiotics’ effectiveness than people who do not use TCIM. 32.6% of all participants reported receiving information in the last 12 months about taking antibiotics only when necessary. 57.2% of all participants, and 66.8% of TCIM users, expressed interest in using homeopathy as a non-antibiotic treatment for common infections (e.g., uncomplicated respiratory tract infection or urinary tract infection).

### Scientific monitoring

ENHANCE includes a quarterly systematic literature search to identify new publications and registered studies on homeopathy and AMR/stewardship. Between December 2023 and May 2026, 39 publications were identified in peer-reviewed journals across RCTs, systematic reviews, pre-clinical studies, observational studies, qualitative studies, agro-homeopathy, case series and research agendas, indicating sustained international research activity in this field.

## ENHANCE: ongoing and planned work

### Systematic reviews for tonsillitis and sinusitis

Building on the acute otitis media foundation, in April 2025, ENHANCE launched systematic reviews examining homeopathy for tonsillitis and sinusitis. These conditions were prioritised based on: (1) high antibiotic prescribing rates despite self-limiting natural history in many cases; (2) preliminary evidence suggesting potential homeopathic effectiveness; and (3) feasibility of conducting future RCTs in community and primary care settings.

Both reviews follow the methodological template established for the otitis media work: PROSPERO pre-registration (CRD42025649244 and CRD42025649258), comprehensive database searches, dual independent screening, Cochrane risk-of-bias assessment, and GRADE evidence certainty rating. Meta-analysis will be conducted where appropriate, with careful attention to heterogeneity across populations, interventions and outcomes (Higgins et al., 2023).

### Core outcome sets and measurement tools

In March 2026, ENHANCE initiated Core Outcome Set projects for tonsillitis and sinusitis. This project follows the same stakeholder-inclusive methodology, ensuring that future RCTs in these conditions use standardised, patient-centred outcome measures.

### Randomised controlled trials/real world evidence studies

ENHANCE also includes the planning and design of large, collaborative primary or community care studies examining homeopathy’s effectiveness for reducing antibiotic use in primary care infections while maintaining acceptable patient outcomes. Planned trials will employ both classical RCT designs and pragmatic designs reflecting real-world primary care practice. Key design features will include, among others, rigorous blinding, intention-to-treat analysis, digital adherence monitoring, health economic evaluation, and nested qualitative studies. Studies are planned for acute otitis media, tonsillitis and sinusitis, contingent on systematic review findings demonstrating sufficient preliminary evidence.

## Discussion and future perspective

The ENHANCE Programme represents the first systematically coordinated international research effort to explore the potential role of homeopathy in antimicrobial stewardship. Overall, the completed work confirms three key points: 1) there is a directional signal favouring antibiotic reduction across various infection types and clinical settings; 2) the necessary methodological infrastructure to produce definitive evidence (including standardised outcomes, pre-registered protocols, and international networks) is now established; and 3) the population using homeopathy and other TCIM alongside conventional healthcare is sufficiently large to make the issue of policy relevance undeniable. What remains is to identify, through adequately powered primary research, whether this directional signal is replicable and clinically meaningful.

### Situating the evidence within the scientific debate

As homeopathy remains one of the most debated areas in medicine, it would be inappropriate to present ENHANCE’s research rationale without acknowledging the contested scientific landscape in which it operates. The rationale for continued investigation is based on a more specific and focused question than the broad debate about effectiveness: namely, whether homeopathy, regardless of its mechanism of action, produces a clinically meaningful reduction in antibiotic prescribing for primary care infections without compromising clinical outcomes. This is an empirical question that warrants testing through adequately powered, methodologically rigorous studies. The consistent positive directional indication across the existing literature does not prove efficacy, but it provides sufficient grounds to properly test the hypothesis. The scientific community is entitled to expect that this test is conducted to the highest standards and ENHANCE is designed to deliver that.

### Integration with one health and AMR strategies

ENHANCE explicitly situates its work within One Health frameworks, recognising that AMR is not a human health problem alone. The majority of global antimicrobial consumption occurs in food-producing animals, and the interdependence of human, animal, and environmental health means that stewardship strategies must operate across all three domains to be effective. The Programme’s connection to the Global Initiative for Traditional Solutions to Antimicrobial Resistance (GIFTS-AMR) Network, comprising seventeen research institutes and six additional organisations across human and veterinary medicine, provides the collaborative infrastructure needed to extend the evidence base beyond primary care into livestock and agricultural settings (Baars et al., 2025).

The Programme’s work aligns with the WHO’s Global Traditional Medicine Strategy 2025–2034 and the Quadripartite One Health Joint Plan of Action 2022–2026 which emphasise evidence generation, regulatory strengthening, and people-centred integration of safe and effective TCIM practices (Food and Agriculture Organization of the United Nations et al., 2022; World Health Organization, 2025).

### Public health relevance

The national representative survey finding that 65.9% of UK adults used TCIM over a 12-month period highlights the relevance of ENHANCE’s research. This is not a marginal patient group. If future trials confirm meaningful antibiotic-sparing effects, the overall population impact of supporting or integrating evidence-based homeopathic care could be significant. However, the survey also raised an equity concern that deserves serious attention: while TCIM use overall was linked to financial difficulty, indicating wide reach across socioeconomic groups through accessible self-care practices, professional TCIM consultation was strongly associated with private health insurance coverage. This suggests that access to practitioner-delivered homeopathy is currently influenced by income and insurance status. If future evidence supports integration into stewardship pathways, implementation models would need to explicitly address this structural inequality, including exploring NHS-accessible delivery models.

### Future perspectives

The immediate priority is to complete and disseminate the ongoing systematic reviews, establishing whether the signal observed in AOM and tonsillitis is consistent across other primary-care infections. These reviews will guide the design and feasibility parameters for future RCTs, with the COS-AOM offering a template for methodological development in each additional condition. Beyond evidence generation, translating findings into healthcare practice will require concurrent investment in implementation science and close collaboration with regulatory bodies, professional organisations and health commissioners, including investigation of NHS-accessible delivery models alongside the clinical evidence base. Expanding into veterinary and agricultural sectors will require adapted outcome frameworks and context-specific feasibility assessments, in close partnership with veterinary research collaborators within GIFTS-AMR (Baars et al., 2025).

A limitation of ENHANCE to date concerns the geographic scope of stakeholder input into its Core Outcome Set, which draws predominantly on high-income, Europe/USA-based clinicians, researchers and patients, even though the underlying systematic reviews included studies from Low- and middle-income country (LMIC) settings worldwide without language restriction. This matters given that AMR burden falls disproportionately on low- and middle-income countries. Future work will include broadening COS stakeholder input and establishing LMIC research partnerships to ensure programme tools and outcome measures are relevant beyond the high-income contexts in which they were initially developed. In many LMICs, traditional medicine practitioners are key health contacts and are interested in community health, making it culturally appropriate to include evidence-based TCIM into local stewardship.

Including LMIC research partners would support WHO’s goals of Universal Health Coverage and equitable access to AMS.

The broader role of TCIM in international antimicrobial stewardship policy requires coordinated, ongoing engagement. Aligning ENHANCE’s research agenda with the WHO Global Traditional Medicine Strategy 2025–2034 and the Quadripartite One Health Joint Plan of Action 2022–2026 creates a policy opportunity that should be actively and responsibly utilised (Food and Agriculture Organization of the United Nations et al., 2022; World Health Organization, 2025). The scale of the AMR crisis, coupled with the fact that a significant proportion of the global population already integrates TCIM into healthcare-seeking behaviour, makes this a question that the research community, policymakers and health systems cannot afford to overlook.

## Conclusion

The ENHANCE Programme has completed the essential groundwork to advance a contested yet significant research question from an initial premise to a thorough investigation. Three key elements are now firmly established: a consistent directional indication favouring reduced antibiotic use across various infection types and clinical environments; an established methodological framework, including standardised outcomes, pre-registered protocols, and an international collaborative network, capable of producing definitive evidence; and a sufficiently large population base that makes the issue undeniably relevant to policy. These foundations collectively justify moving forward with sufficiently powered primary research to evaluate whether the directional trend is reproducible and clinically meaningful.

The Programme’s completed work to date has identified promising though inconclusive evidence in acute otitis media, produced the first internationally agreed Core Outcome Set for that condition, and documented significant TCIM use across diverse socioeconomic groups. It has also surfaced a structural equity concern that demands explicit attention. Any pathway towards integration into stewardship practice must, from the outset, be designed around NHS-accessible delivery models, so that evidence of benefit translates into equitable rather than stratified care.

ENHANCE situates this work within the One Health and global AMR policy contexts to which it genuinely belongs. The ongoing systematic reviews, forthcoming RCTs, and real-world evidence studies represent the next phase of an evidence-generation pipeline that is now structurally sound. The central question of whether homeopathy can meaningfully contribute to reducing antibiotic use at a population level, with symptom control and equitable access, is one that the research community and health systems cannot responsibly leave unanswered in the context of a deepening global antimicrobial resistance crisis.

## Data Availability

The original contributions presented in the study are included in the article/supplementary material. Further inquiries can be directed to the corresponding author.

## Appendix

**Table:** APPENDIX 1 List of ENHANCE collaborators.

Dr Rachel Perry	Translational Health Sciences, Bristol Medical School, University of Bristol, Bristol, UK
Dr Alyson L. Huntley	Centre for Academic Primary Care, Bristol Medical School, University of Bristol, Bristol, UK
Professor Nai Ming Lai	School of Medicine, Faculty of Health and Medical Sciences, Taylor’s University, Kuala Lumpur, Malaysia
Dr Michael Teut	Institute for Social Medicine, Epidemiology and Health Economics, Charité Universitätsmedizin Berlin, Germany
Professor David D. Martin	Tübingen University Children’s Hospital, Tübingen University, Tübingen, Germany; Institute of Integrative Medicine, Department of Medicine, University of Witten/Herdecke, Witten, Germany
Professor Thomas Ostermann	Department for Psychology, Faculty of Health, University of Witten/Herdecke, Witten/Herdecke, Germany
A/professor Henrik Szőke	Department of Integrative Medicine, Faculty of Health Sciences (ETK), University of Pécs, Pécs, Hungary
Ms Rachel Roberts	Homeopathy Research Institute, London, UK
Professor Jon Adams	Australian Research Consortium in Complementary and Integrative Medicine, Faculty of Health, Southern Cross University, Lismore, Australia
Professor Amie Steel	Australian Research Consortium in Complementary and Integrative Medicine, Faculty of Health, Southern Cross University, Lismore, Australia National Centre for Naturopathic Medicine, Faculty of Health, Southern Cross University, Lismore, Australia
Dr Hope Foley	Australian Research Consortium in Complementary and Integrative Medicine, Faculty of Health, Southern Cross University, Lismore, Australia National Centre for Naturopathic Medicine, Faculty of Health, Southern Cross University, Lismore, Australia
Dr Tristan Carter	Australian Research Consortium in Complementary and Integrative Medicine, Faculty of Health, Southern Cross University, Lismore, Australia National Centre for Naturopathic Medicine, Faculty of Health, Southern Cross University, Lismore, Australia

## References

[B1] Albrecht H. Schutte A. (1999). Homeopathy versus antibiotics in metaphylaxis of infectious diseases: a clinical study in pig fattening and its significance to consumers. Altern. Ther. Health Med. 5, 64–68. 10484832

[B2] Antimicrobial Resistance Collaborators (2022). Global burden of bacterial antimicrobial resistance in 2019: a systematic analysis. Lancet 399, 629–655. doi: 10.1016/S0140-6736(21)02724-0 37500506

[B3] Baars E. W. Weiermayer P. Szoke H. P. van der Werf E. T. (2025). The introduction of the global traditional, complementary, and integrative healthcare (TCIH) research agenda on antimicrobial resistance and its added value to the WHO and the WHO/FAO/UNEP/WOAH 2023 research agendas on antimicrobial resistance. Antibiotics (Basel) 14. doi: 10.3390/antibiotics14010102 39858387 PMC11762681

[B4] Camerlink I. Ellinger L. Bakker E. J. Lantinga E. A. (2010). Homeopathy as replacement to antibiotics in the case of Escherichia coli diarrhoea in neonatal piglets. Homeopathy 99, 57–62. doi: 10.1016/j.homp.2009.10.003 20129177

[B5] Commission of the European Communities (1997). Homeopathic Medicinal Products, Commission report to the European Parliament and the Council on the application of Directives 92/73 and 92/74 Brussels, 1997. Brussels: Commission of the European Communities

[B6] Dombrowsky C. Klein S. D. Wurtenberger S. Baumgartner S. Tournier A. L. (2025). Mapping the theories and models on the mode of action of homeopathy: a scoping review. J. Integr. Complement Med. 31, 937–945. doi: 10.1089/jicm.2024.1007 40495556

[B7] Ebert F. Staufenbiel R. Simons J. Pieper L. (2017). Randomized, blinded, controlled clinical trial shows no benefit of homeopathic mastitis treatment in dairy cows. J. Dairy Sci. 100, 4857–4867. doi: 10.3168/jds.2016-11805 28342609

[B8] Elvers K. T. Wilson V. J. Hammond A. Duncan L. Huntley A. L. Hay A. D. . (2020). Antibiotic-induced changes in the human gut microbiota for the most commonly prescribed antibiotics in primary care in the UK: a systematic review. BMJ Open 10, e035677. doi: 10.1136/bmjopen-2019-035677 32958481 PMC7507860

[B9] Faedo L. Matias C. Verdi R. Wright J. Rayns F. Kretzschmar A. . (2024). The use of mineral dynamised high dilutions for natural plant biostimulation; effects on plant growth, crop production, fruit quality, pest and disease incidence in agroecological strawberry cultivation. Biol. Agric. Horticulture 40, 267–287. doi: 10.1080/01448765.2024.2396894 37339054

[B10] Food and Agriculture Organization of the United Nations United Nations Environment Programme World Health Organization World Organisation for Animal Health (2022). One Health Joint Plan of Action (2022–2026). Working Together for the Health of Humans, Animals, Plants and the Environment (Rome: Food and Agriculture Organization of the United Nations (FAO)).

[B11] Friese K. H. Kruse S. Ludtke R. Moeller H. (1997). The homoeopathic treatment of otitis media in children--comparisons with conventional therapy. Int. J. Clin. Pharmacol. Ther. 35, 296–301. 9247843

[B12] Gaertner K. Ulbrich-Zurni S. Baumgartner S. Walach H. Frass M. Weiermayer P. (2023). Systematic reviews and meta-analyses in homeopathy: recommendations for summarising evidence from homeopathic intervention studies (Sum-HomIS recommendations). Complement Ther. Med. 79, 102999. doi: 10.1016/j.ctim.2023.102999 37898390

[B13] Grimaldi-Bensouda L. Begaud B. Rossignol M. Avouac B. Lert F. Rouillon F. . (2014). Management of upper respiratory tract infections by different medical practices, including homeopathy, and consumption of antibiotics in primary care: the EPI3 cohort study in France 2007-2008. PloS One 9, e89990. doi: 10.1371/journal.pone.0089990 24646513 PMC3960096

[B14] Hamre H. J. Glockmann A. von Ammon K. Riley D. S. Kiene H. (2023). Efficacy of homoeopathic treatment: systematic review of meta-analyses of randomised placebo-controlled homoeopathy trials for any indication. Syst. Rev. 12, 191. doi: 10.1186/s13643-023-02313-2 37805577 PMC10559431

[B15] Hektoen L. Larsen S. Odegaard S. A. Loken T. (2004). Comparison of homeopathy, placebo and antibiotic treatment of clinical mastitis in dairy cows - methodological issues and results from a randomized-clinical trial. J. Vet. Med. A. Physiol. Pathol. Clin. Med. 51, 439–446. doi: 10.1111/j.1439-0442.2004.00661.x 15610489

[B16] Higgins J. P. T. Thomas J. Chandler J. Cumpston M. Li T. Page M. J. . (2023). Cochrane Handbook for Systematic Reviews of Interventions, Version 6.4 (Updated August 2023) (London, UK: Cochrane).

[B17] Ivemeyer S. Walkenhorst M. Klocke P. Heil F. Oser S. . (2008). Auswirkungen einer zweijährigen Bestandesbetreuung von Milchviehbeständen hinsichtlich Eutergesundheit, Antibiotikaeinsatz und Nutzungsdauer [Effects of a two-year dairy herd health management programme on udder health, use of antibiotics and longevity. Schweiz. Arch. Tierheilkd 150, 499–505. doi: 10.1024/0036-7281.150.10.499 18821509

[B18] Keller D. Sundrum A. (2018). Comparative effectiveness of individualised homeopathy and antibiotics in the treatment of bovine clinical mastitis: randomised controlled trial. Vet. Rec. 182, 407. doi: 10.1136/vr.104555 29374099 PMC5890642

[B19] Klocke P. Ivemeyer S. Butler G. Maeschli A. Heil F. (2010). A randomized controlled trial to compare the use of homeopathy and internal teat sealers for the prevention of mastitis in organically farmed dairy cows during the dry period and 100 days post-calving. Homeopathy 99, 90–98. doi: 10.1016/j.homp.2009.12.001 20471611

[B20] Lejri I. Grimm A. Trempat P. Boujedaini N. Eckert A. (2025). Gelsemium low doses protect against serum deprivation-induced stress on mitochondria in neuronal cells. J. Ethnopharmacol. 336, 118714. doi: 10.1016/j.jep.2024.118714 39181289

[B21] Mathie R. T. Lloyd S. M. Legg L. A. Clausen J. Moss S. Davidson J. R. . (2014). Randomised placebo-controlled trials of individualised homeopathic treatment: systematic review and meta-analysis. Syst. Rev. 3, 142. doi: 10.1186/2046-4053-3-142 25480654 PMC4326322

[B22] Mathie R. T. Ramparsad N. Legg L. A. Clausen J. Moss S. Davidson J. R. . (2017). Randomised, double-blind, placebo-controlled trials of non-individualised homeopathic treatment: systematic review and meta-analysis. Syst. Rev. 6, 63. doi: 10.1186/s13643-017-0445-3 28340607 PMC5366148

[B23] Ostermann T. Park A. L. De Jaegere S. Fetz K. Klement P. Raak C. . (2021). Cost-effectiveness analysis for SilAtro-5-90 adjuvant treatment in the management of recurrent tonsillitis, compared with usual care only. Cost Eff. Resour. Alloc 19, 60. doi: 10.1186/s12962-021-00313-4 34538271 PMC8451093

[B24] Page M. J. McKenzie J. E. Bossuyt P. M. Boutron I. Hoffmann T. C. Mulrow C. D. . (2021). The PRISMA 2020 statement: an updated guideline for reporting systematic reviews. BMJ 372, n71. doi: 10.1136/bmj.n71 33782057 PMC8005924

[B25] Palm J. Kishchuk V. V. Ulied A. Fernandez J. P. De Jaegere S. Jong M. C. . (2017). Effectiveness of an add-on treatment with the homeopathic medication SilAtro-5-90 in recurrent tonsillitis: an international, pragmatic, randomized, controlled clinical trial. Complement Ther. Clin. Pract. 28, 181–191. doi: 10.1016/j.ctcp.2017.05.005 28779928

[B26] Pannek J. Pannek-Rademacher S. Jus M. S. Wollner J. Krebs J. (2019). Usefulness of classical homeopathy for the prophylaxis of recurrent urinary tract infections in individuals with chronic neurogenic lower urinary tract dysfunction. J. Spinal Cord Med. 42, 453–459. doi: 10.1080/10790268.2018.1440692 29485355 PMC6718136

[B27] Perry R. Huntley A. L. Lai N. M. Martin D. D. van der Werf E. T. (2024). The effectiveness of homeopathy in relieving symptoms and reducing antibiotic use in patients with otitis media: a systematic review and meta-analysis. Heliyon 10, e39174. doi: 10.1016/j.heliyon.2024.e39174 39640837 PMC11620159

[B28] Perry R. H. A.L. Lai N. M. Roberts E. R. van der Werf E. T. (2026). Homeopathy for tonsillitis: a systematic review. Eur. J. Integr. Med. 85. doi: 10.1016/j.eujim.2026.102666 38826717

[B29] Pouwels K. B. Dolk F. C. K. Smith D. R. M. Robotham J. V. Smieszek T. (2018). Actual versus 'ideal' antibiotic prescribing for common conditions in English primary care. J. Antimicrob. Chemother. 73, 19–26. doi: 10.1093/jac/dkx502 29490060 PMC5890776

[B30] Prasad R. (2007). Homoeopathy booming in India. Lancet 370, 1679–1680. doi: 10.1016/S0140-6736(07)61709-7 18035598

[B31] Sonnenschein-Swanson L. Baur-Bernhardt S. Käsbohrer A. Doherr M. G. Meemken D. Sommer M.-A. . (2026). Management, production, infection events, and antimicrobial use on 25 commercial Turkey farms in Germany (2019–2021)—a descriptive analysis. Poultry 5. doi: 10.3390/poultry5020019 30654563

[B32] Stub T. Kristoffersen A. E. Overvag G. Jong M. C. Musial F. Liu J. (2022). Adverse effects in homeopathy. A systematic review and meta-analysis of observational studies. Explore (NY) 18, 114–128. doi: 10.1016/j.explore.2020.11.008 33303386

[B33] Stub T. Musial F. Kristoffersen A. A. Alraek T. Liu J. (2016). Adverse effects of homeopathy, what do we know? A systematic review and meta-analysis of randomized controlled trials. Complement Ther. Med. 26, 146–163. doi: 10.1016/j.ctim.2016.03.013 27261996

[B34] Taylor J. A. Jacobs J. (2014). Homeopathic ear drops as an adjunct in reducing antibiotic usage in children with acute otitis media. Glob. Pediatr. Health 1, 2333794X14559395. doi: 10.1177/2333794X14559395 27335917 PMC4804695

[B35] Ucker A. Baumgartner S. Martin D. Jager T. (2022). Critical evaluation of specific efficacy of preparations produced according to European Pharmacopeia Monograph 2371. Biomedicines 10. doi: 10.3390/biomedicines10030552 35327354 PMC8944999

[B36] UUK Department of Health and Social Care (2019). UK 5-Year Action Plan for Antimicrobial Resistance 2019 to 2024 (London: DHSC).

[B37] UK Department of Health and Social Care (2024). Confronting Antimicrobial Resistance 2024 to 2029 (London: DHSC).

[B38] van der Werf E. T. Foley H. Carter T. Roberts R. Adams J. Steel A. (2026). Traditional, integrative and complementary medicine use in the UK population: results of a nationally representative cross-sectional survey. BMJ Open 16, e104334. doi: 10.1136/bmjopen-2025-104334 41545039 PMC12815132

[B39] van der Werf E. T. Perry R. Ostermann T. Szoke H. Huntley A. L. (2025). A core outcome set for acute otitis media (COS-AOM) for primary and community care studies. BMC Prim Care 26, 134. doi: 10.1186/s12875-025-02821-1 40296004 PMC12036145

[B40] Varanasi R. Bhalerao R. Sumithran P. Rompicherla K. G. Singh P. Ramteke S. S. . (2025). A comparative randomized controlled trial of homeopathy versus allopathy in acute otitis media and its recurrence in children. Complement Med. Res. 32, 110–121. doi: 10.1159/000542800 39864417

[B41] Venekamp R. P. Sanders S. L. Glasziou P. P. Rovers M. M. (2023). Antibiotics for acute otitis media in children. Cochrane Database Syst. Rev. 11, CD000219. doi: 10.1002/14651858.CD000219.pub5 37965923 PMC10646935

[B42] Werner C. Sobiraj A. Sundrum A. (2010). Efficacy of homeopathic and antibiotic treatment strategies in cases of mild and moderate bovine clinical mastitis. J. Dairy Res. 77, 460–467. doi: 10.1017/S0022029910000543 20822562

[B43] Williamson P. R. Altman D. G. Bagley H. Barnes K. L. Blazeby J. M. Brookes S. T. . (2017). The COMET handbook: version 1.0. Trials 18, 280. doi: 10.1186/s13063-017-1978-4 28681707 PMC5499094

[B44] Witt C. M. Bluth M. Albrecht H. Weisshuhn T. E. Baumgartner S. Willich S. N. (2007). The *in vitro* evidence for an effect of high homeopathic potencies--a systematic review of the literature. Complement Ther. Med. 15, 128–138. doi: 10.1016/j.ctim.2007.01.011 17544864

[B45] World Health Organization (2025). Global Traditional Medicine Strategy 2025–2034 (Geneva: World Health Organization). 10.2471/BLT.25.293414 PMC12578523 41180270

[B46] World Health Organization (2015). Global Action Plan on Antimicrobial Resistance (Geneva: WHO).

[B47] Wustrow T. P. Otovowen Study Group (2004). Alternative versus conventional treatment strategy in uncomplicated acute otitis media in children: a prospective, open, controlled parallel-group comparison. Int. J. Clin. Pharmacol. Ther. 42, 110–119. 15180172

